# Supplementary material for: Statistical inferences for type-II hybrid censoring data from the alpha power exponential distribution
Source: PLoS One. 2021 Jan 20;16(1):e0244316. doi: 10.1371/journal.pone.0244316 (PMC7817043; doi:10.1371/journal.pone.0244316)
Supplement: Attachment — Submitted filename: response to reviewers.docx [file pone.0244316.s001.docx]

**Authors Response**

Authors response to reviewer comments on article “Statistical Inferences for Type-II Hybrid Censoring Data from the Alpha Power Exponential Distribution”. We would like to thank the reviewers for their great effort to make this work more complete. We have carefully read all of the comments and have made necessary modifications to our revised manuscript. We believe the presentation of the paper has improved, thanks to your comments and contributions. We have done all the changes in the revised draft. The reviewers’ comments and our respective responses are listed below.

| No. | Remarks of Reviewer#1 | Response from authors | Modifications Done |
| --- | --- | --- | --- |
| 1 | Reference “ A multivariate random parameters Tobit model for analyzing highway crash rate by injury severity. Accident Analysis and Prevention, 2017, 99: 184-191”. | Added | Done |
| 2 | Reference “Spatial joint analysis for zonal daytime and nighttime crash frequencies using a Bayesian bivariate conditional autoregressive model. Journal of Transportation Safety and Security, 2020, 12(4): 566-585.” | Added | Done |
| 3 | Some directions for future research are suggested to draw in the Conclusion Section. | Its deserve to study in future the inferences on APE parameters under a balanced two-sample type-II progressive censoring scheme. | Added to conclusion |

| No. | Remarks of Reviewer#2 | Response from authors | Modifications Done |
| --- | --- | --- | --- |
| 1 | Reference “Investigating the Differences of Single- and Multi-vehicle Accident Probability Using Mixed Logit Model, Journal of Advanced Transportation, 2018, UNSP 2702360. | Added | Done |
